# Supplementary material for: skDER and CiDDER: two scalable approaches for microbial genome dereplication
Source: Microb Genom. 2025 Jul 10;11(7):001438. doi: 10.1099/mgen.0.001438 (PMC12245536; doi:10.1099/mgen.0.001438)
Supplement: Uncited Supplementary Material 1. [file mgen-11-01438-s001.pdf]

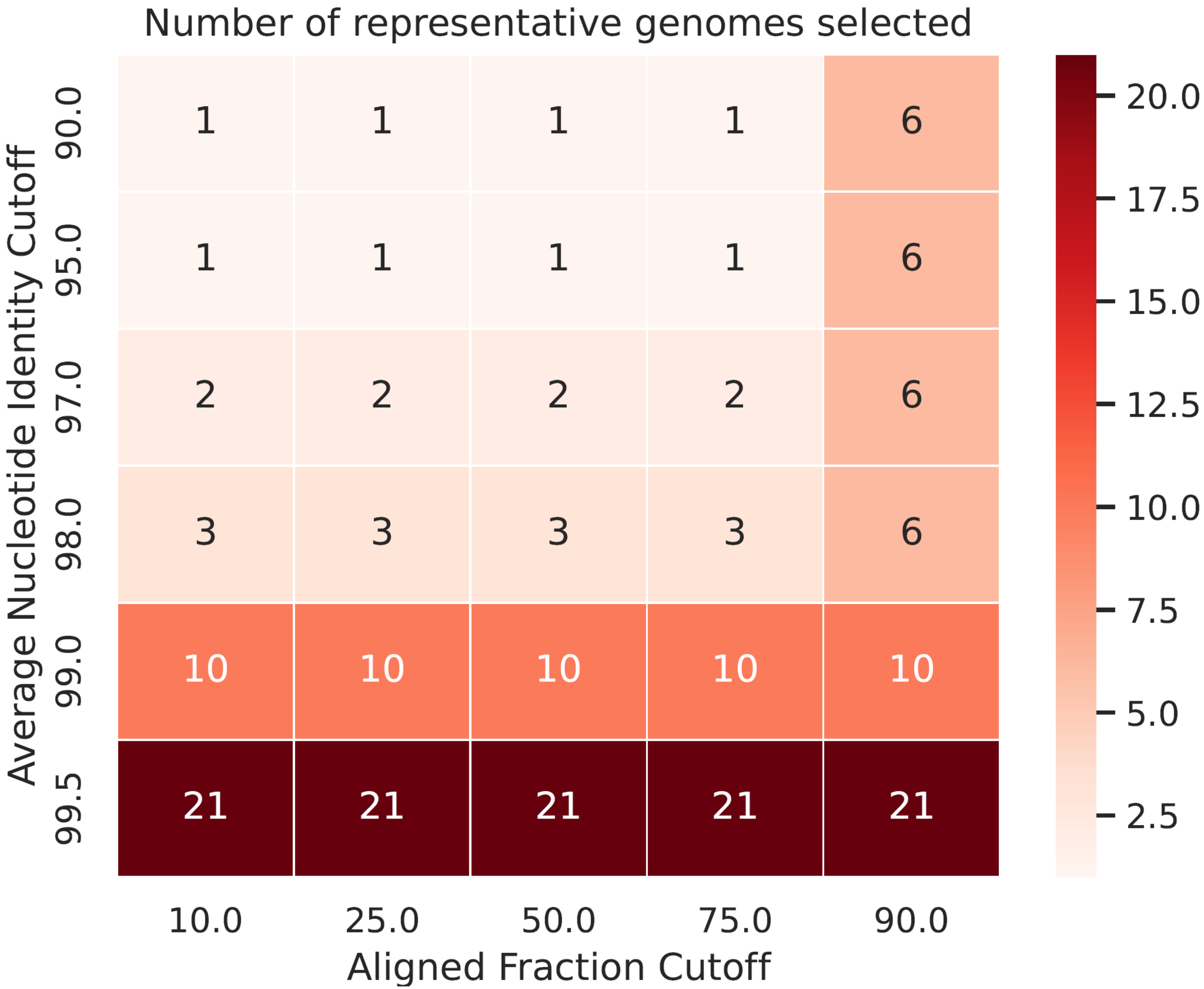

Figure S1: Assessing the number of representatives from using different combinations of ANI and AF. An example visual produced by skDER when run in the "greedy" mode and the option "--test-cutoffs" was specified to test for the effect of using different combinations of ANI and AF on the final number of representative genomes selected.

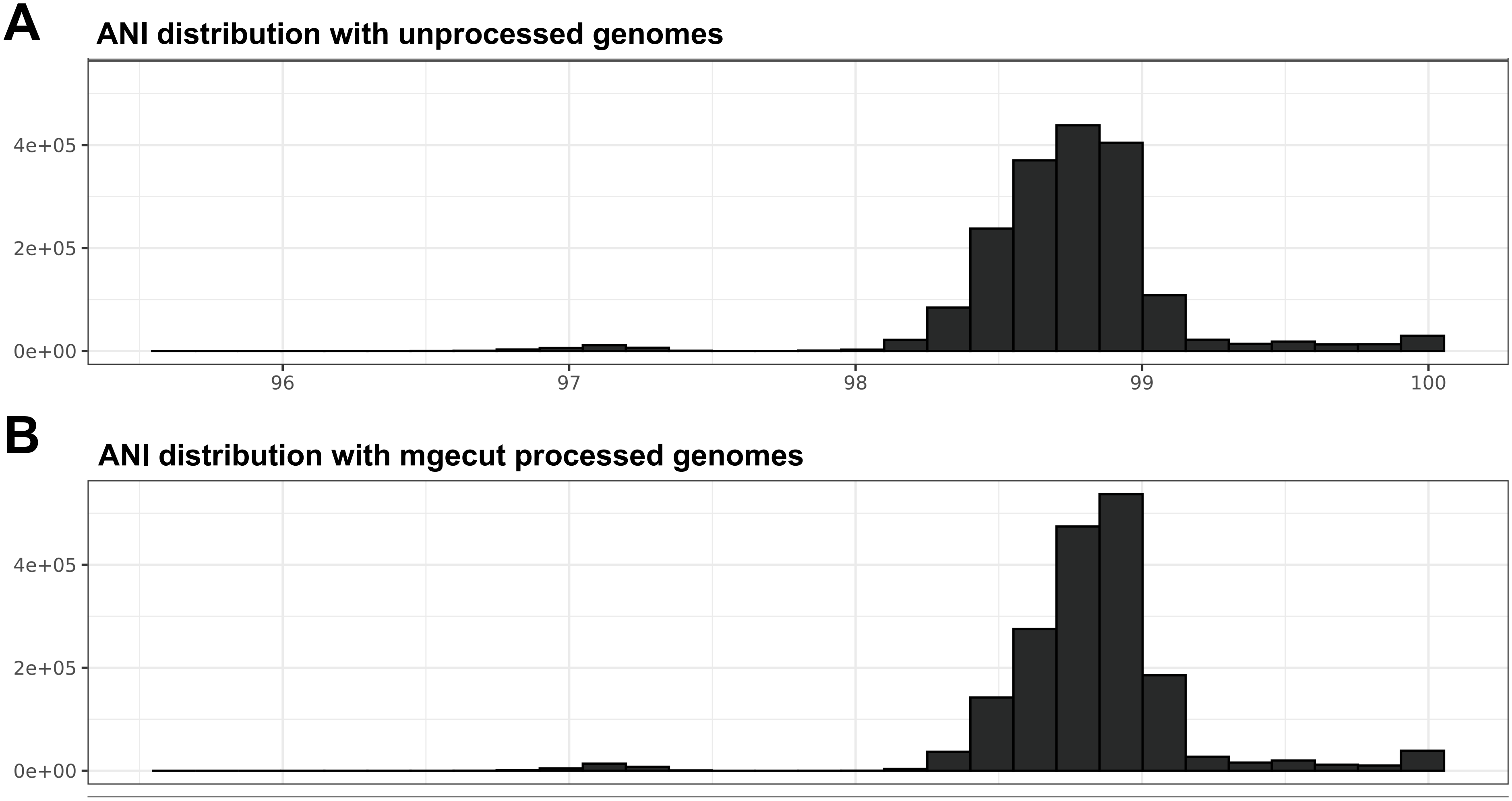

Figure S2: The intra-species ANI gap determined by Rodriguez-R et al. 2024 is still present after MGE filtering. The distribution of ANI estimates between pairs of 1,902 *E. faecalis* genomes which: **A)** are unprocessed/unfiltered and **B)** have been filtered for MGEs using mgecut. The intra-species ANI gap from approximately 99.2% to 99.8% identity reported on by Rodriguez-R et al. 2024 is observed regardless of whether MGEs are filtered from genomes.

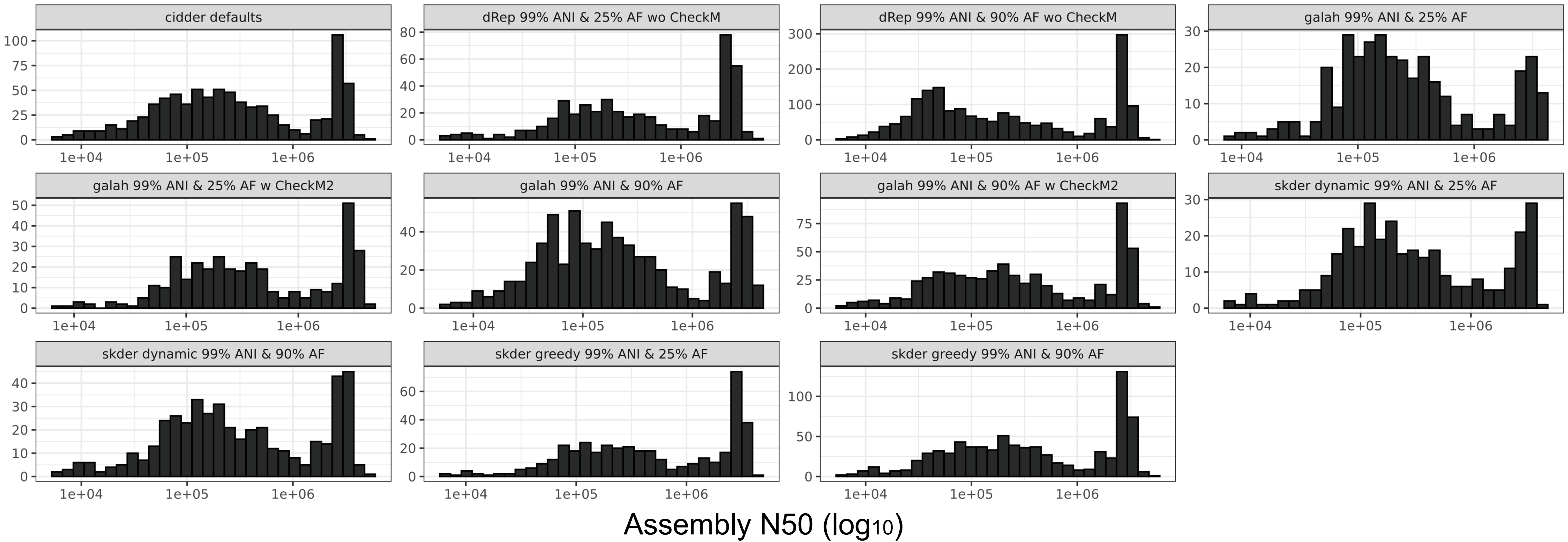

Figure S3: N50 distributions of representative genomes from different dereplication software. Distributions for assembly N50 are shown for representative genomes selected by different dereplication software.

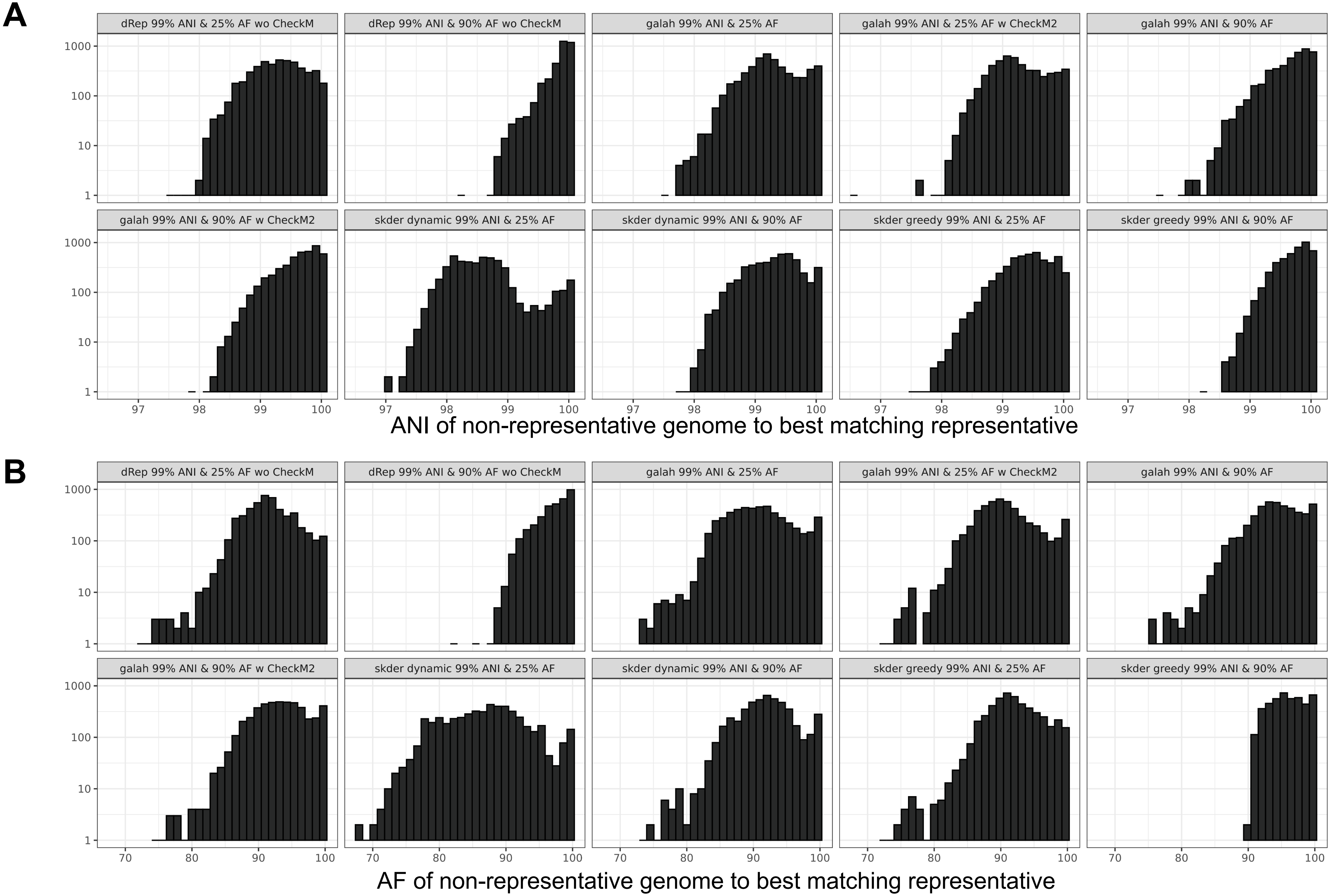

Figure S4: Differences in distributions for ANI and AF of non-representative genomes to their best matching representative genomes based on using different dereplication software. Histograms represent distributions for ANI and AF between non-representative genomes and their best matching representative genome based on skani estimates for different dereplication software. The best matching representative genome was selected per non-representative genome based on a metric equivalent to the product of the ANI and non-representative AF. Note, for the secondary clustering performed in skDER, a different approach is used to find the best matching representative genome for each non-representative genome.
