# Supplementary material for: skDER and CiDDER: two scalable approaches for microbial genome dereplication
Source: Microb Genom. 2025 Jul 10;11(7):001438. doi: 10.1099/mgen.0.001438 (PMC12245536; doi:10.1099/mgen.0.001438)
Supplement: Uncited Supplementary Material 2. [file mgen-11-01438-s002.pdf]

Supplementary Text

Salamzade, Kottapalli, and Kalan, 2025

skDER Greedy:

- Download or process input genomes.
- Compute and create a tsv linking each genome to their N50 assembly quality metric (*N50*[g]).
- Compute ANI and AF using skani triangle to get a tsv "edge listing" between pairs of genomes (with filters applied based on ANI and AF cutoffs).
- Run through "edge listing" tsv on first pass and compute connectivity (*C*[g]) for each genome - how many other genomes it is similar to at a certain threshold
  - Only consider a genome as connected to a focal genome if they share an ANI greater than the --percent_identity_cutoff (default of 99% before v1.3.0 and 99.5% after) and the comparing genome exhibits an AF greater than the --aligned_fraction_cutoff (default of 90% before v1.3.0 and 50% after) to the focal genome (is sufficiently representative of both the core and auxiliary content of the focal genome).
- Run through "N50" tsv and compute the score for each genome: *N50*[g]**C*[g] = *S*[g] and write to new tsv where each line corresponds to a single genome, the second column corresponds to the *S*[g] computed, and the third column to connected genomes to the focal genome.
- Sort resulting tsv file based on *S*[g] in descending order and use a greedy approach to select representative genomes if they have not been accounted for as a connected genome from an already selected representative genome with a higher score.

skDER Dynamic:

- Download or process input genomes.
- Compute and create a tsv linking each genome to their N50 assembly quality metric (*N50*[g]).
- Compute ANI and AF using skani triangle to get a tsv "edge listing" between pairs of genomes (with filters applied based on ANI and AF cutoffs).
- Run through "edge listing" tsv on first pass and compute connectivity (*C*[g]) for each genome - how many other genomes it is similar to at a certain threshold.
- Run through "N50" tsv and store information.
- Second pass through "edge listing" tsv and assess each pair one at a time keeping track of a singular set of genomes regarded as redundant:
  - if (*AF*[g_1] - *AF*[g_2]) >= parameter --max_af_distance_cutoff (default of 10%), then automatically regard corresponding genome of max(*AF*[g_1], *AF*[g_2]) as redundant.
  - else calculate the following score for each genome: *N50*[g]**C*[g] = *S*[g] and regard corresponding genome for min(*S*[g1], *S*[g2]) as redundant.
- Second pass through "N50" tsv file and record genome identifier if they were never deemed redundant.

CiDDER:

- Download or process input genomes.
- Predict proteins using pyrodigal.
- Comprehensive clustering of all proteins using CD-HIT
- Select genome with the most number of distinct protein clusters as the initial representative.
- Iteratively add more representative genomes one at a time, selecting the next based on maximized addition of novel protein clusters to the current representative set.
- End addition of representative genomes if one of three criteria are met: (i) Next genome adds less than X number of distinct protein clusters (X is by default 0), (ii) over Y% of the total distinct protein clusters across all genomes are found in the so-far selected representative genomes (Y is by default 90%), or (iii) over Z% of the total distinct multi-genome protein clusters across all genomes are found in the so-far selected representative genomes (Z is by default 100%). Thus, by default, only Y is used for representative genome selection.
